# New Trends in Robotics for Agriculture: Integration and Assessment of a Real Fleet of Robots

**DOI:** 10.1155/2014/404059

**Published:** 2014-03-30

**Authors:** Luis Emmi, Mariano Gonzalez-de-Soto, Gonzalo Pajares, Pablo Gonzalez-de-Santos

**Affiliations:** ^1^Centre for Automation and Robotics (UPM-CSIC), Arganda del Rey, 28500 Madrid, Spain; ^2^Department of Software Engineering and Artificial Intelligence, Faculty of Informatics, University Complutense of Madrid, 28040 Madrid, Spain

## Abstract

Computer-based sensors and actuators such as global positioning systems, machine vision, and laser-based sensors have progressively been incorporated into mobile robots with the aim of configuring autonomous systems capable of shifting operator activities in agricultural tasks. However, the incorporation of many electronic systems into a robot impairs its reliability and increases its cost. Hardware minimization, as well as software minimization and ease of integration, is essential to obtain feasible robotic systems. A step forward in the application of automatic equipment in agriculture is the use of fleets of robots, in which a number of specialized robots collaborate to accomplish one or several agricultural tasks. This paper strives to develop a system architecture for both individual robots and robots working in fleets to improve reliability, decrease complexity and costs, and permit the integration of software from different developers. Several solutions are studied, from a fully distributed to a whole integrated architecture in which a central computer runs all processes. This work also studies diverse topologies for controlling fleets of robots and advances other prospective topologies. The architecture presented in this paper is being successfully applied in the RHEA fleet, which comprises three ground mobile units based on a commercial tractor chassis.

## 1. Introduction

In the last twenty years, specialized sensors (machine vision, global positioning systems (GPS) real-time kinematics (RTK), laser-based equipment, and inertial devices), actuators (hydraulic cylinder, linear, and rotational electrical motors), and electronic equipment (embedded computers, industrial PC, and PLC) have enabled the integration of many autonomous vehicles, particularly agricultural robots [1–5]. These autonomous/semiautonomous systems provide accurate positioning and guidance in the working field, which makes them capable of conducting precision agricultural tasks if equipped with the proper implements (agricultural tools or utensils). Those implements (variable application rates of fertilizers or sprays, mechanical intrarow weed control, and seed planters) are also being automated with the same types of sensors and actuators used in autonomous vehicles (GPS, machine vision, range finders, etc.) [6–11]. Thus, when integrating a given vehicle and a particular implement, many sensors and/or actuators are duplicated and, worst of all, a central, external computer must be used to coordinate the arrangement: vehicle and implement. Minimizing the hardware of the vehicle-implement system is essential for commercializing reliable, efficient, and cost-competitive agricultural machinery [12]. Thus, devising a simple controller for both vehicle and implement would facilitate reliability, efficiency, and competitiveness.

Many research groups are developing specialized autonomous applications for agriculture that will be operative in the coming years [13–15], but many others are also aiming to operate a group of vehicles under unified control. This is the emergent concept of fleets of robots, which represents a step forward in agriculture. The associated theoretical foundations fleets of robots have been investigated recently [16, 17], but the first applications for agriculture are currently under development [18, 19]. For this purpose, the concept of reducing redundant devices coordinating different, heterogeneous systems by using a central, external computer is prominent.

Fleets of robots can provide many advantages [20–23]: using a group of robots cooperating with each other to achieve a well-defined objective is an emerging and necessary concept to achieve the application of autonomous systems in daily agricultural tasks. The implementation of complex and expensive systems will be attractive for high-value crops for which smart machines can replace extensive and expensive repetitive labor. However, for a robotic agricultural application, considerable information must be processed, and a wide number of actuation signals must be controlled, which may present a number of technical drawbacks. Thus, an important limitation is that the number of total devices (e.g., sensors, actuators, and computers/controllers) increases according to the number of fleet units, and thus the mean time between failures decreases drastically because a failure in one robot component causes the entire fleet to be out of order. This decrease in the time between failures significantly influences fleet reliability, which is of paramount importance for the application of automated systems to real tasks and, in particular, to agriculture.

To achieve a flexible, reliable, and maintainable fleet of autonomous mobile robots for agricultural tasks, the system architecture (involving sensors, actuators, and the computers performing the algorithms) for both the vehicle navigation system and the operation of the implement must be robust, simple, and modular. One of the most important tasks in a control configuration design is the selection of the number and type of sensors, actuators, and computers. These components constitute the basis for the design of the architecture and are very difficult to decrease in number because the processes of perceiving and actuating cannot be avoided; however, these sensors and actuators are typically handled by independent controllers, specifically, commercial off-the-shelf (COTS) sensors such as LIDARs and vision systems. However, computers are sufficiently flexible to share resources and improve the robustness of the system.

**Figure 1:**
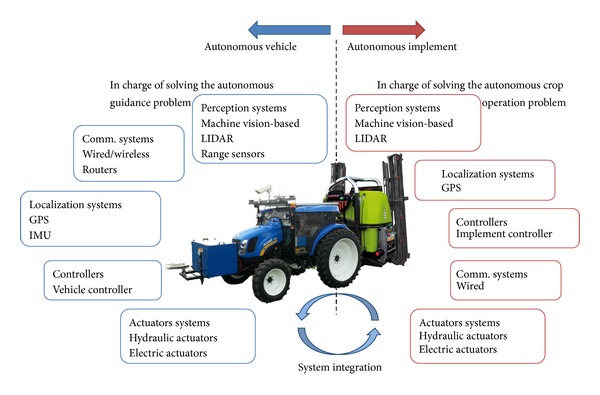
Main systems comprising a current autonomous agricultural application and some examples of sensor and actuation systems normally found in this type of application.

In fully autonomous agricultural systems, several actions must be executed simultaneously to ensure effective application as well as safety (including the system, the crop field, and external elements, e.g., human supervisors). Absolute or relative localization in the field, obstacle and interesting element detection, communication with external users or with other autonomous units, autonomous navigation or remote operation, and site-specific applications are some of these specific actions that, all together, compose a fully autonomous agricultural system. This system can be divided into two main subsystems (see Figure 1): the autonomous vehicle and the autonomous implement. The autonomous vehicle, such as a modified commercial tractor, specialized platform, or small vehicle, guides the agricultural system in a crop field for the purpose of executing a crop operation (e.g., harvesting, hoeing, and weed control), which will be accomplished by the autonomous implement. Given the complexity of the assignment, a large number of specialized sensors and actuators are required to fulfill the given task in the given environment.

For each individual system presented in Figure 1, intensive research activities have been documented in the literature that intend to solve both the autonomous guidance problem and the autonomous crop operation problem individually.  Table 1 presents selected examples of efforts to solve the autonomous guidance problem, and Table 2 presents some works focused on solving the autonomous crop operation problem, indicating the application for which they were developed and the main sensor system used.

A few attempts to establish a fully autonomous agricultural system by integrating an autonomous vehicle and an autonomous implement can be found in the scientific literature. One of the most important examples is the work conducted in Denmark by Nørremark et al. [14]. These authors developed a self-propelled and unmanned hoeing system for intrarow weed control comprising an autonomous tractor [24] and a cycloid hoe [25] linked via a hydraulic side-shifting frame attached to the rear three-point hitch of the tractor. In this system, the autonomous tractor follows a predefined route parallel to the crop rows and turns at the end of the rows, the side-shift frame adjusts its lateral position depending on predefined waypoints, and the cycloid hoe controls the tines to avoid contact with crop plants. Both the vehicle and the implement are controlled independently according to a predefined mission. However, some sensorial systems are replicated; for example, there is one GPS for the vehicle guidance and another for the side-shifting and cycloid hoe control systems.

**Figure 2:**
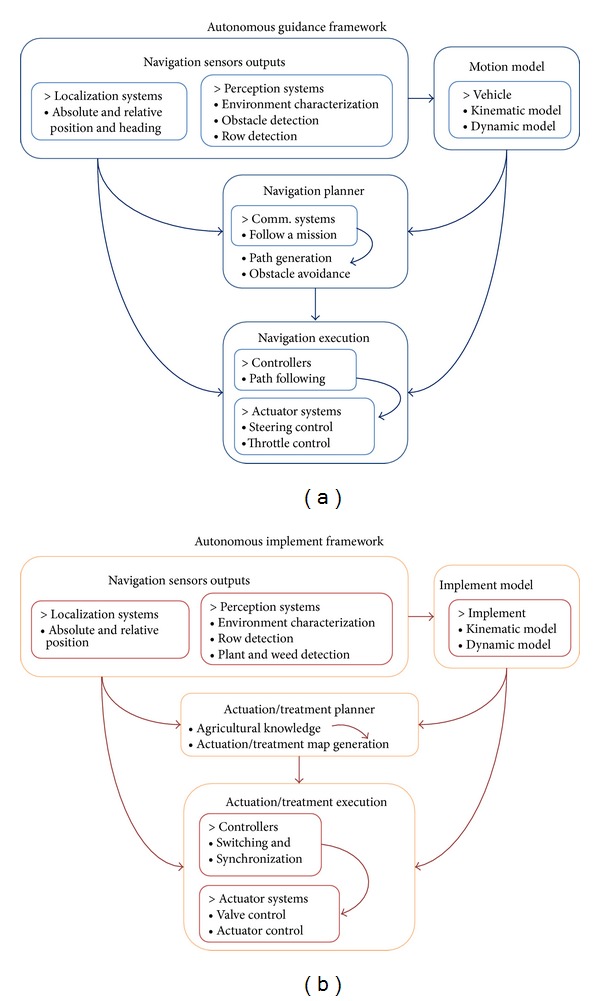
General frameworks of a fully autonomous crop operation. (a) Basic elements of agricultural vehicle guidance systems. (b) Basic elements of autonomous implements.

Some authors agree that, in general terms, the framework of an agricultural autonomous guidance system mainly consists of four subtasks: sensor acquisition, modeling, planning, and execution [3, 26]. Based on this generalization, Figure 2(a) presents a simplified framework for agricultural guidance in which the outputs of each subtask are highlighted. Following this framework, and based on a review of the research activities in autonomous crop operations over the last fifteen years, we can construct an analogy and present a general framework of agricultural autonomous implements (see Figure 2(b)).

For each framework, we can identify some similarities in (a) the usage of sensors and actuators, (b) the flow data general scheme, and (c) the specific subtask that uses the sensors and actuators. For example, the use of machine vision in both frameworks is commonly applied for row detection to localize and adjust the relative position of the vehicle/implement depending on the environment; the use of the GPS in both frameworks is commonly applied for absolute localization to follow a predefined route or for the application of a specific treatment in a specific location.

## 2. Problem Approach

To obtain a fully autonomous agricultural system, the aforementioned two general frameworks must be merged in an architecture (hardware and software) that shares the sensorial system and the planning methods for both the autonomous guidance and the autonomous treatment application. This task must be performed with the objective of reducing the amount of hardware while maintaining the required performance. This architecture must be capable of integrating different sensor and actuation systems developed by diverse research groups as well as different types of commercial equipment. Furthermore, it must be flexible and integrate several standard communication protocols that are common in high-tech agricultural applications [27]. A modular architecture to provide convenient settings of the interfaces between the sensors and devices and proper organization of the perception, processing, and actuation of these types of systems are required due to the large variety of available technologies.

Thus, as a first step, this paper focuses on the design of a proper structure for mobile autonomous vehicles collaborating as a fleet of robots in agricultural tasks. Hardware reliability, truly plug-and-play features, and programmability are essential for efficient agricultural vehicles and, consequently, for competent fleets of robots, but modularity, expandability, ergonomics, maintenance, and cost are also of paramount importance to increase the number of prospective real applications in agriculture.

The aforementioned basic features are considered in the proposed configuration; however, other features are also discussed in the following sections with the primary aim of providing manufacturers of agricultural machinery with solutions for automating new developments, particularly in precision agriculture, an emerging area demanding robust and efficient solutions.

**Figure 3:**
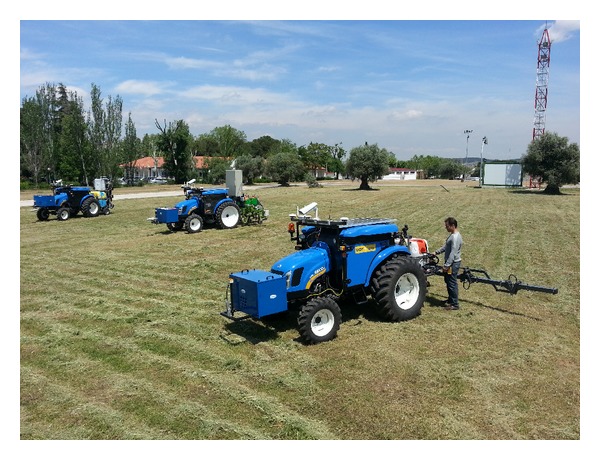
The RHEA fleet (ground mobile units and implements).

The work presented in this paper has been conducted within the RHEA project, a FP7 program project granted by the European Commission. RHEA is focused on the development of a new generation of vehicles for effective chemical and mechanical management of a large variety of crops to minimize the use of agricultural inputs to decrease environmental pollution while improving crop quality and safety and reducing production costs [21]. To accomplish this aim, RHEA is conducting research in (a) advanced perception systems to detect and identify crop status, including crop row detection, and (b) innovative actuation systems to apply fertilizers and herbicides precisely as well as to remove or eliminate weeds directly. Additional research is focused on the development of (c) a fleet of small, safe, reconfigurable, heterogeneous, and complementary mobile units to guarantee the application of the procedures to the entire operation field. This scientific activity must be complemented with technical developments in (d) novel communication and location systems for robot fleets, (e) enhanced simulation systems and collaborative graphic user interfaces, and (f) pioneering fuel cells to build clean and efficient energy sources (see Figure 3).

To accomplish these overall objectives, we have developed the structure presented in this paper, which is organized as follows. First, the architecture of an autonomous system is introduced in Section 3; in Section 4, we collect the requirements for agricultural fleets of robots; different topologies for fleets of robots are discussed in Section 5; finally, Section 6 presents some results, followed by conclusions in Section 7.

## 3. Structuring a Fully Autonomous Agricultural System

The first idea that comes to mind to structure a fully autonomous agricultural application is to take two operational subsystems such as those presented in the previous section (one autonomous vehicle and one autonomous implement) and put them to work together. This demands a communication mechanism between the autonomous vehicle and the autonomous implement in the form of a Main Controller, that is responsible for merging the desired behavior of each individual subsystem into a single behavior that treats the fully autonomous agricultural system like a robot unit. Thus, the whole system can be broken down into three main modules: vehicle, implement, and controller.

### 3.1. The Vehicle

The vehicle is the module in charge of ensuring the motion behavior of the implement (absolute position and orientation) and must adapt both the type of crop and the type of operation on the crop. Normally, the vehicle carries or tows the implement and therefore provides the necessary energy to the implement as well. Thus, the vehicle must include mechanical adaptors (three-point hitch) to fulfill agricultural standards, electrical generators, and hydraulic pumps. These specific subsystems are provided by commercial agricultural vehicles, and thus adapting a commercial agricultural tractor to configure an autonomous vehicle is easier and more efficient than developing an agricultural robot from scratch. This also allows the developers to advance system integration and testing while avoiding other time-consuming activities such as chassis design, manual assembly, testing, and vehicle homologation, for instance. These modifications drastically increase vehicle reliability by using long-term tested items (engine, braking, steering and transmission systems, housing, etc.) while decreasing time until availability. The safety, robustness, and efficiency of the system must also be considered when structuring the entire autonomous system.

The final selected vehicle for the RHEA project was a CNH Boomer-3050 (51 hp—37.3 KW, 1200 kg), whose restructured and empty cabin was used to contain the computing equipment for the perception, communication, location, safety, and actuation systems. In addition, some systems require the placement of specific elements outside the cabin: vision camera, laser, antennas (GPS and communications), and emergency bottoms. This overall equipment can be classified into the following subsystems:

- a Weed Detection System to detect weed patches that relies on machine vision;
- a crop row detection system to help steer the vehicle based on machine vision;
- a laser range finder to detect obstacles in front of the mobile units;
- communication equipment linking the operator station, the mobile units, and the user portable devices;
- a two-antenna global positioning system to locate/orientate the vehicle in the mission field;
- an inertial measurement unit (IMU) to complement the GPS data and enable improved vehicle positioning;
- a vehicle controller in charge of computing the steering control law, throttle, and braking for path tracking purposes. Steering, throttle, clutching, and braking are the mechanisms normally provided by modern vehicles via a CAN bus;
- a central controller as a decision-making system responsible for gathering information from all perception systems and computing the actions to be performed by the actuation components;
- an additional energy power supply based on a fuel cell, which is monitored by the central controller.

**Figure 4:**
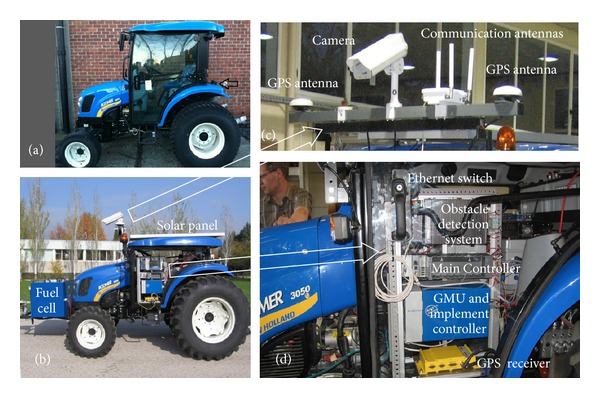
(a) Initial commercial tractor, (b) final RHEA mobile unit, (c) external equipment onboard the mobile units, and (d) internal equipment distribution inside the mobile unit's cabin.

Figures 4(a) and 4(b) illustrate the original and modified Boomer T3050, respectively. The latter image shows the reduced cabin, the fuel cell, the solar panel placed on top of the robot, the antenna bar, and the equipment distribution inside the cabin. These two last elements are magnified in Figures 4(c) and 4(d).

### 3.2. Implements

The implement is a device designed to perform an action on the crop, such as herbicide and pesticide booms and mechanical and thermal weed removers. The nozzles and burners found on implements are normally operated independently to focus the actuations according to precision agricultural principles. Some of those elements have positioning devices to improve treatments. PLCs and computers are used to control those independent elements and coordinate actions with the vehicle.

The RHEA project has developed three different implements so far.

**Figure 5:**
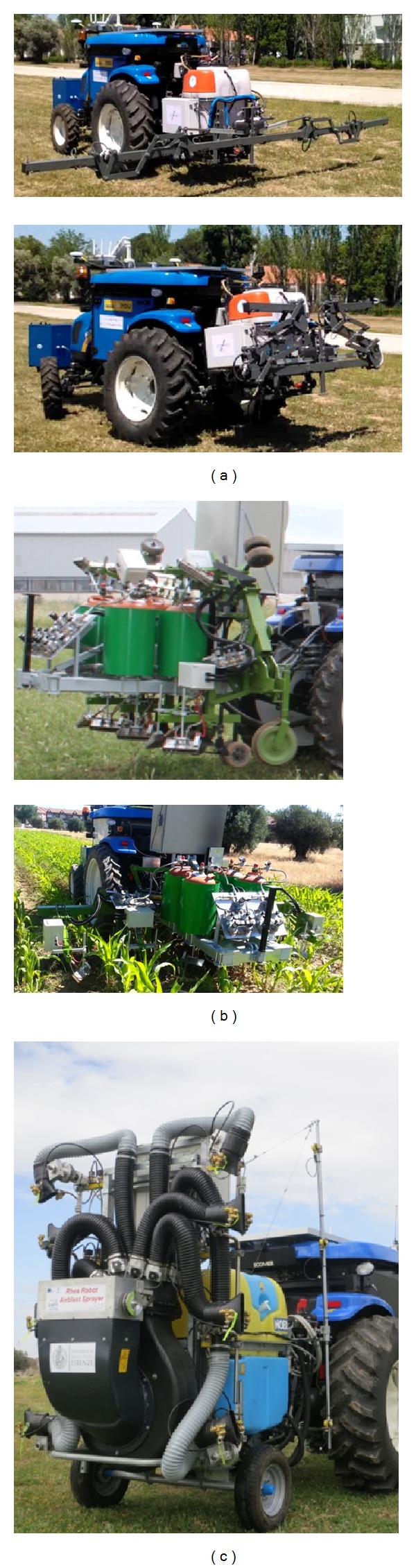
Implements controlled by the RHEA system: (a) boom sprayer, (b) flame hoe, and (c) canopy sprayer.

*A boom sprayer* [28] for herbicide application in cereals (see Figure 5(a)) consists of a 5.5 m boom containing 12 nozzles separated by 0.5 m and exhibiting independent actuation. The implement is carried by the vehicle and contains two herbicide tanks (200 L and 50 L, resp.), the contents of which can be mixed to apply different treatments. The flow of herbicide through the nozzles as well as the boom folding/unfolding device is controlled by the vehicle's Main Controller.

*A mechanical-thermal machine* [29] is for weed control (see Figure 5(b)) in flame-resistant crops such as maize, onion, and garlic. This system consists of four couples of burners attached to a main frame that tackles four consecutive crop rows. The implement is towed by the vehicle, which is also responsible for controlling the relative lateral position of the implement with respect to the vehicle's position. The flame intensity of each burner is a function of the amount of weeds detected by the Weed Detection System based on machine vision. That amount is expressed as the percentage of the area covered by weeds in every area unit—typically 0.25 m × 0.25 m. The vehicle's controller is also in charge of the folding/unfolding device.

*An airblast sprayer* [30] for pesticide application in olive trees (see Figure 5(c)) consists of two vertical booms with four nozzles each. The lower and upper nozzles are oriented by stepper motors based on the information provided by a set of ultrasound sensors, one per nozzle, with the aim of maximizing the amount of pesticide applied to the canopies. The vehicle passively tows the implement, which contains all of the sensorial systems required for the aforementioned application.

The aim of this section is simply to illustrate the large number of different types of sensors and actuators used in these implements. Thus, the detailed aspects of these designs are considered outside of the scope of this paper.

### 3.3. Main Controller

The Main Controller is in charge of steering the vehicle accurately, coordinating the actions of the vehicle, and maintaining communication with the operator. In addition, the Main Controller integrates a large number of subsystems, such as those mentioned in Section 3.1. Integrating different systems based on diverse communication technologies, operating systems, and programming languages leads to questions about the organization of the hardware and software architecture, which can be centralized or distributed, open-source or commercial development software, among others. These options have advantages and disadvantages that can be found in any technical material on the topic. A distributed architecture is based on several computers running different applications on similar or dissimilar operating systems. The computers are connected by a communication network or point-to-point communication links exhibiting very well-known features as well as a few shortcomings derived from its maintenance cost in terms of updates and security, the number of different operating systems and programming languages to be handled, time-consuming management needs, and network delays in communicating data, which may impair the real-time features of the system.

Apart from considering the advantages and limitations of both configurations, the optimum choice depends on the specific application, that is, the number of sensors; the number and type of peripherals; the number of different computers, including operating systems and languages used; the required computing power; and the real-time requirement, among other factors. This task is relatively easy to perform in a closed requirement system, that is, a system in which we know the exact number of subsystems and their features. However, in agriculture, the number of different system configurations, the available commercial devices, and custom-made equipment make the selection of the optimum configuration a difficult task, particularly due to the different operating systems and programming languages.

The best solution, as in other engineering fields, could be to use a hybrid architecture featuring centralized and distributed characteristics capable of integrating new systems when possible and permitting the connectivity of the complex system by using distribution features, such as Ethernet networks and a CAN bus, among others.

In the last ten years, developers of robotic systems, particularly universities and research centers, have been attempting to consolidate and package robotic frameworks as open-source software available to the entire robotic community. Examples of these frameworks are MOOS [31], PLAYER [32], CARMEN [33], and ROS [34], among others, which are essentially network-based communication architectures that allow diverse nodes or applications to communicate and interact with each other. These applications are packages developed by other research groups or by the users, and they are commonly used in the academic community and research centers and commonly applied in service robots. Currently, the most popular open-source operating system for robots is ROS (Robot Operating System), a software platform comprising a large collection of open-source libraries and tools that was initiated in 2007 for the development of robot software and provides the functionality of an operating system on a heterogeneous computer cluster. This system provides standard operating system services (hardware abstraction, device control low-level message passing between processes, implementation of commonly used functions, and package management). ROS is the reference in many research and academic developments because it is free and powerful, but it is released under the terms of the Berkeley Software Distribution licenses, a family of permissive free software licenses that imposes minimal restrictions on the redistribution of covered software, complicating its application to systems for commercial exploitation [35].

Although packaging a robotic framework facilitates the integration of systems from different providers with very dissimilar features, it also leads to problems related to revealing the expertise (making the software code available to others may cause replication and loss of financial benefit) and loss of income through sales (revenue must be gained through support agreements and OEM customization). Recently, some have suggested that ROS should be locked down, protected, and commercialized [36] to monetize industrial and service robots [37].

## 4. Identifying Architecture Requirements for Agricultural Fleets

### 4.1. Fully Autonomous Agricultural System Requirements

As presented in Sections 2 and 3, an important aspect of structuring an architecture for a fully autonomous agricultural system (vehicle and implement) is the reduction of the amount of sensors and actuators of the entire system, which constitute the basis for the design of the hardware architecture. However, decreasing the amount of devices for sensing and acting is a difficult task because these components are needed for the correct operation of the system.

Analyzing the two general frameworks presented in Figure 2 reveals that some tasks for guidance and actuation require similar sensorial systems and similar information processing, particularly the tasks of localization, perception, and planning. Furthermore, in a fully autonomous system, instead of having two processes for each of the aforementioned tasks, which would replicate hardware and software elements, these similar tasks can be merged to reduce the amount of specialized hardware.

When merging the tasks of each individual subsystem, the problem of resource assignment and synchronization arises. In addition, the vehicle and implement move according to different reference frames, but a general behavior of the fully autonomous agricultural system must be determined as a part of the general mission of the entire fleet of robots.

Another key element of the hardware architecture is the ability to allow diverse vehicle and implement configurations to enable a fleet of heterogeneous robots to execute diverse crop operations at the same time. To achieve this capability, the hardware architecture must be modular to allow diverse sensory and actuating elements to be rapidly and easily replaced, installed, and configured, thus modifying a small part of the fully autonomous system to enable diverse crop operations. The link between sensors and actuators relies on the computer system.

Given this preliminary discussion, agricultural fleets of robots should rely on the following elements.

- A hybrid computing system consisting of a central, powerful, truly real-time, multitasking computer with fast network communication features to connect different peripherals.
- The central computer should have a large family of real plug-and-play hardware modules including both reliable wired and wireless communication modules.
- The central computer should provide capabilities to facilitate running software developed for different platforms and in different languages.
- Simple and powerful connections to external libraries and third-party tools must be included.
- The development tools must allow diverse programming languages for different applications and domain experts in different disciplines (e.g., agronomists and roboticists) and must permit multidisciplinary use, for example, a graphic programming system.
- The central computer should allow a wide variety of data acquisition and embedded control devices, which tightens software-hardware integration.
- The central computer should be ruggedized to operate in harsh conditions and allow intrinsic parallelism: multicore-ready design and support for different hardware acceleration technologies: DSPs (Digital Signal Processing), FPGAs (Field-Programmable Gate Array), and GPUs (Graphic Processing Units) as coprocessors.
- The central computer must have the capability to execute and solve complex algorithms in real-time using real-world external signals (A/D).
- The entire architecture must be able to transition easily from academics to industry, ensuring protection of property rights.

Many of these features are fulfilled by the new family CompactRIO-9082 (cRIO-9082: 1.33 GHz dual-core Intel Core i7 processor, 32 GB nonvolatile storage, 2 GB DDR3 800 MHz RAM), high-performance integrated systems commercialized by National Instruments Corporation, whose equipment has already been used in some unmanned road vehicles [38, 39] and autonomous agricultural vehicles [13]. The selected system offers a powerful stand-alone and networked execution for deterministic, real-time applications. This hardware platform contains a reconfigurable Field-Programmable Gate Array (FPGA) for custom timing, triggering, and processing and a wide array modular I/O for any application requirement. This system is designed for extreme ruggedness, reliability, and I/O flexibility, which is appropriate for the integration of different sensorial and actuation systems in precision agriculture autonomous applications.

Furthermore, LabVIEW (Laboratory Virtual Instrumentation Engineering Workbench) is a graphical programming environment used to measure, test, and control different systems by using intuitive graphical icons and wires resembling a flowchart. This environment facilitates integration with thousands of hardware devices, provides hundreds of built-in libraries for advanced analysis and data visualization, and can help prototype applications with FPGA technology.

These hardware/software features ensure (a) performance: equipment reliability and robustness in harsh environments; (b) compatibility: a large list of modules is available for peripherals, including serial and parallel standard communications; (c) modularity/expandability: a computer-based system includes a set of configurable modules that allow the system to grow according to the application needs; (d) developer community: the increasing number of LabVIEW users sharing their experiences and developments in the form of packages or functions freely and openly through blogs and forums; and (e) cost: while the investment in NI hardware and software is initially high, profitability must consider the reduction of the development in person-months as well as the reduction of the hardware when manufacturing medium-to-large prototype series by using products such as the Single-Board RIO.

Based on the previous analysis of both hardware and software features, we have proposed the aforementioned system as the Main Controller of the RHEA robot fleet [40]. Key features in the selection of the present controller were the capabilities of configuring a minimum hardware and assuring a short period for software development. These features allow the developers to focus on the implementation of new algorithms and on the integration of sensors and actuators.

### 4.2. Fleet Management Topology Requirements

Once the architecture requirements for the implementation of a fully autonomous agricultural system are defined, it is necessary to define the requirements for the fleet of robot, which comprises several robot units as described in the previous sections, oriented to agricultural applications. Basically, a fleet is a set of independent mobile units that must be coordinated somehow and interfaced with (a) the environment or workspace; (b) with each other; and (c) with an operator, at given instants. In robotic agriculture, the workspace normally is well known: the dimensions of the field or set of fields are well demarcated; the field is planted or needs to be planted with a specific crop; the boundaries and fixed obstacles are well known; and the areas where the vehicles can travel are well determined. In addition, coordinated motion in this workspace involves relatively small teams of both similar (e.g., tractors with sprayers) and/or heterogeneous vehicles, (e.g., harvester and truck), depending on the application and the final goal. Each of these situations leads to diverse solutions of the coordinated motion problem. In some applications, where two or more vehicles must constantly cooperate (e.g., autonomous harvesting), motion coordination between nearby vehicles is more critical to ensure path accuracy, dead distance, time, fuel, or other efficiency criteria than in other applications in which each vehicle performs a defined and repetitive task without cooperation (e.g., transplanting and weed control). Some attempts have been made to solve the coordinated motion problem in the form of conceptual farming system architectures [41–45], including some that have put cooperation between robots in agricultural tasks into practice [46, 47].

However, given the workspace characteristics and the well-defined general objective of the fleet, many authors agree that a central planner running on an external computer that knows each of the elements involved in the agricultural application and is capable of readjusting the parameters and assignments is necessary for optimal development of the general agricultural task. However, depending on the type of agricultural application for which the fleet is configured, each autonomous unit could have a greater or lesser ability to replan its own subtasks. Conceptual examples can be found in [48–50].

Even if the workspace is well defined, safety is an important factor that affects the fleet composition. The vehicles should be in frequent communication with the external computer to provide data about current status and operation, and a human operator must be in constant supervision. The operator must be present at some instants: mission configuration, mission start, and mission stop or suspension, among others. Thus, an operator interface is essential.

**Figure 6:**
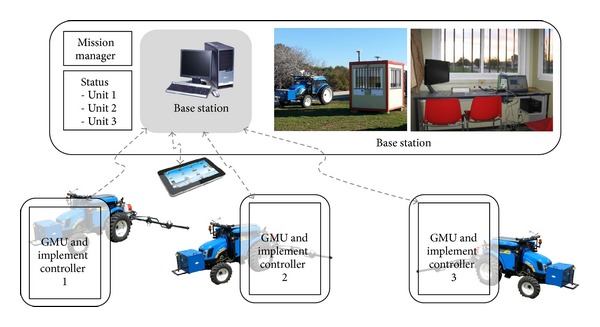
General schema of the fleet of robot topology for the RHEA project.

Based on these requirements, the topology of the fleet of robots defined for the RHEA project was a central-external computer located in a base station (BS) for planning, supervising, and allowing the user to access a full interface, in addition to a user portable device (UPD) that allows the user to approach the units to maintain control and supervision of the fleet (see Figure 6). In this topology, a master external computer connected to the fleet units through a wireless communication system runs a mission manager (mission planner and mission supervisor) that sends commands to (and receives data from) the fleet mobile units.

## 5. Implementation of the Proposed Main Controller: The Evolution of the RHEA Computing System

**Figure 7:**
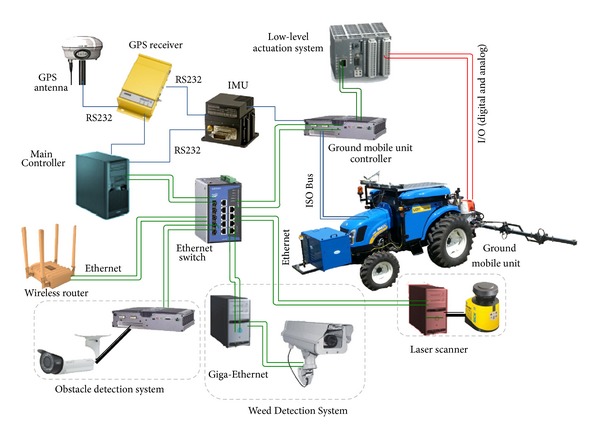
General scheme of the hardware architecture for the autonomous mobile robot in the RHEA project.

The computing system onboard the mobile units must communicate with a large number of subsystems, such as those specified in Section 3.1, which are based on computers running different operating systems (e.g., Windows, Linux, and QNX) and software modules developed in different languages (C++, NET, Python, etc.). The first solution was to connect all subsystems through an Ethernet network and use a computer as a central controller [27]. This initial solution is depicted in Figure 7. The Main Controller is connected to the peripherals through either a serial line or an Ethernet network (802.3 Local Area Network) via an Ethernet switch, which requires a Network Manager running on a computer connected to the Ethernet switch, normally the Main Controller.

**Figure 8:**
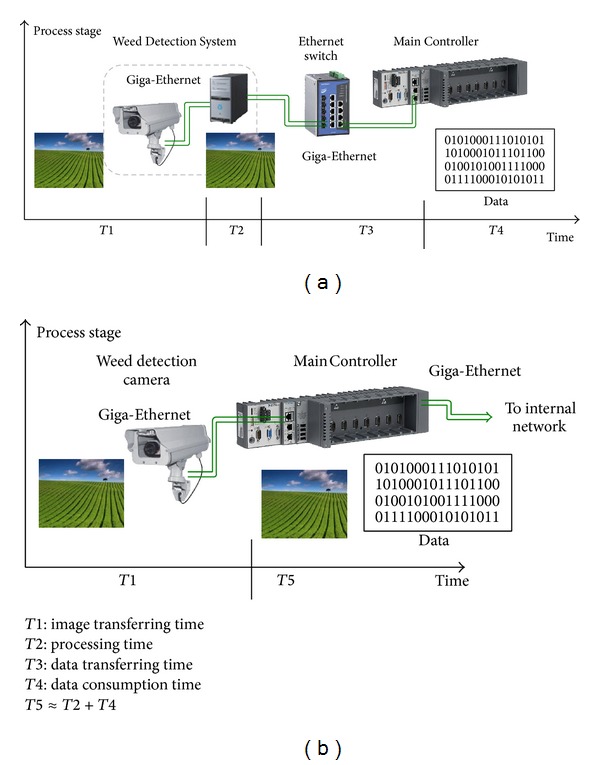
Comparison of the distributed approach (a) and the centralized approach (b) in the Weed Detection System regarding the use of resources, information availability, and communication time.

The first step toward centralization consisted of integrating the Weed Detection System (WDS) into the Main Controller. The vision camera is GigE Vision standard compliant (global camera interface standard developed using the Gigabit Ethernet communication protocol framework for transmitting high-speed video and related control data), and the Main Controller has two Gigabit Ethernet ports. This allows for a direct interface between the camera and the Main Controller using the functionalities provided by LabVIEW for configuration and acquisition, avoiding the development of new drivers and eliminating the vision computer. This solution is illustrated in Figure 8.

**Figure 9:**
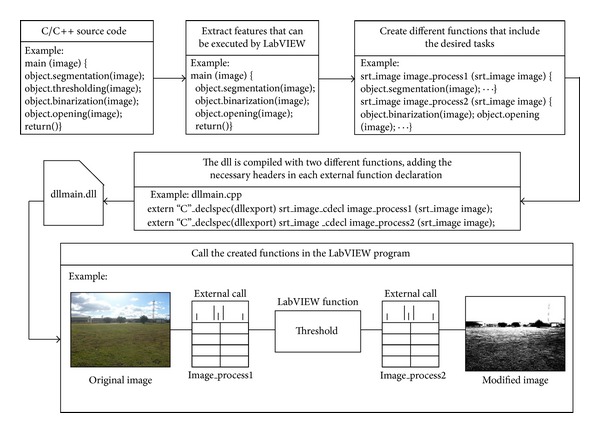
Example of the procedure for calling external code in LabVIEW using DLLs.

Two major problems arose at that time. The first was reusing the acquisition software implemented in C++; the second was to assess the Main Controller running the vision algorithms as an additional charge. The first problem was solved by using the LabVIEW connectivity with third-party tools, which allows the programmers to call external scientific libraries in the form of C code, DLLs (Dynamically Linked Library), and code interface nodes (CINs), which include C code compiled into LabVIEW nodes. The specific solution consisted of converting the vision code developed in the C++ language for Windows 7 into a DLL. This DLL can be loaded into the Main Controller and its functions can be called within the LabVIEW environment. One of the important steps in creating a compatible DLL is the detection and substitution of pieces of code that may have problems during the execution, such as system calls. This problem can occur when an external code developed for other operating systems (in this case Windows 7) is called by the LabVIEW Real-Time Operative System (LabVIEW RTOS) and attempts to access some kernel libraries. This may generate conflicts, and therefore it is recommended that this practice should be avoided as much as possible. Once the source code is adapted for execution in the LabVIEW RTOS, it must be packaged in one or different C language functions following the procedure defined in Figure 9.

There are several advantages of running the real-time features of the algorithms in the same computer (see Figure 8).

- By eliminating the vision computer (WDS computer) and implementing the execution of the weed detection task in the Main Controller, the deterministic performance is increased, which removes an intermediate non-real-time OS (Windows) and an extra Ethernet network link.
- By receiving the information directly from the camera, other processes that are being executed in the Main Controller can rapidly access the images by sharing the same memory space.
- By integrating the acquisition and processing algorithms in the Main Controller, the information about the weed distribution is rapidly shared with other processes that require it, which decreases the communication time and increases the real-time response. While this integration generates a problem of interprocess communication by shared memory, it is compensated (in this particular case) by eliminating the communication between different machines. Because there is no need to share images with other computers, the development of drivers for these tasks is eliminated.

A second strategy to improve the centralization of the RHEA system is to unify the two vision systems: the Weed Detection System (WDS) and the obstacle detection System (ODS). Both systems use similar image-capture mechanisms and image-processing algorithms, which can be integrated into the same computer to save hardware resources. The software, which is written in the C++ language for the Linux operating system, is converted to a DLL following the procedure described for the WDS (see Figure 9). The main problem with this configuration is the lack of real parallelism in the execution of the algorithms, which increases the computing time. However, this increase is compensated by the elimination of the delay in the information flow from the ODS to the Main Controller through the Ethernet. Analogous to the integration process of the WDS into the Main Controller, Figure 8 shows that, in the proposed architecture, the time T3 will be eliminated in the data flow of the ODS in comparison with the original schema. In the proposed architecture, the camera acquires an image and sends it via the Ethernet to the Main Controller in charge of both processing the image and making the decision. By contrast, with the original schema, the ODS information must pass through more network elements, increasing transmission time.

Some advantages of integrating the two vision systems are as follows.

- The application requires only one camera, which reduces the amount of hardware and relevant equipment (the vision fields of both cameras are similar).
- The Main Controller allocates the same memory space to share information between the two vision processes (which can take advantage of the processed image to be used by the two different processes).
- The two processes can be executed in parallel.

One of the further developments and benefits of this integration is the improvement of the performance in an obstacle detection task in real-time applications by fusing the camera and the laser information. Integrating the sensor acquisition methods and the fusion method in the same computer increases the reliability of deadline compliance, data correlation, and synchronization compared to the original scheme. However, if the data acquisition, both with the camera and the laser, is not performed on the decision-making computer, the nondeterministic features of the Ethernet will reduce the real-time capabilities, expanding the timeframe and producing synchronization problems.

**Figure 10:**
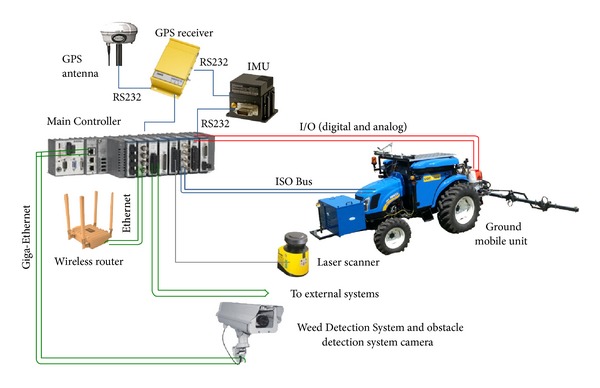
Prospective hardware configuration of the RHEA system.

These are two examples of possible system centralization of complex subsystems; however, other subsystems can be centralized in a simpler way by using the plug-and-play features (e.g., Ethernet communication through WLAN modules and switches; laser and inertial measurement units through RS-232 serial modules; industrial communication buses through CAN bus; ISO modules, low-level actuation system through analog and digital I/O modules; etc.).  Figure 10 shows the final system scheme, which includes the main external sensorial components.

**Figure 11:**
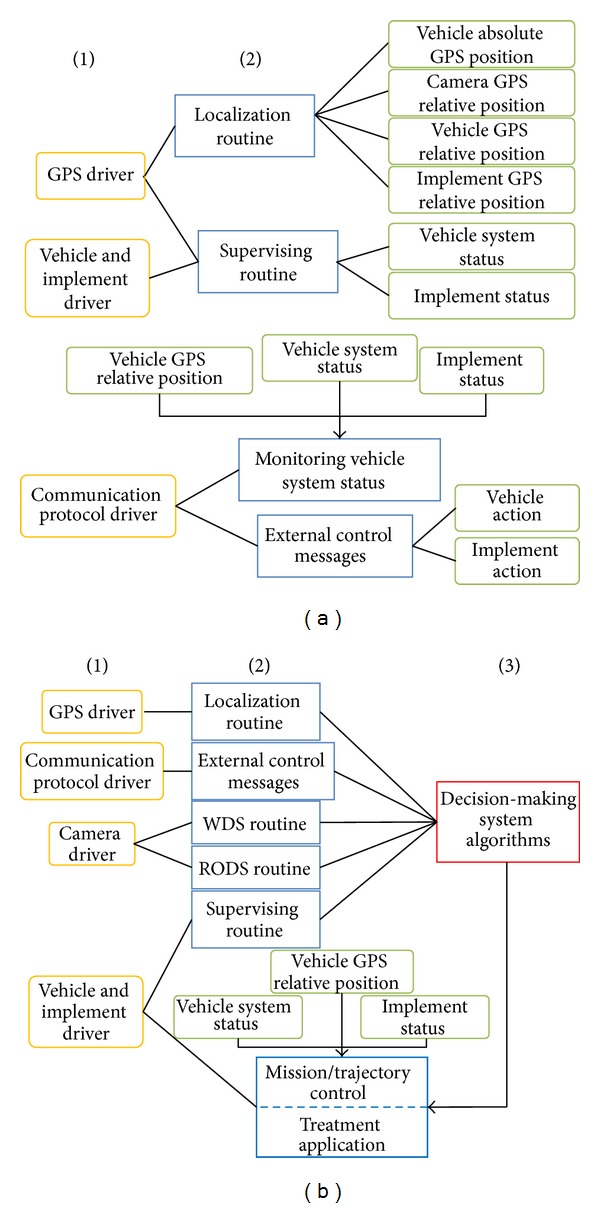
General diagram of the high-level decision-making system indicating three levels of main subsystems, their outputs, and their interactions with other subsystems. (a) Principal outputs (green boxes) of the lower subsystems. (b) Flow between sensory systems and control systems and navigation process execution.

Using this basic controller and taking advantage of the LabVIEW features, we have defined a simple software architecture to connect all subsystems to the main module in charge of making decisions (high-level decision-making system).  Figure 11 illustrates a general schematic diagram in which the following three different software levels are defined.

1. The first level, represented by yellow boxes, consists of driver modules that allow communication with the various sensors, actuators, and other elements of the system (e.g., external user interface).
2. The second level, represented by blue boxes, consists of several modules in charge of interpreting, generating, and/or merging information from the lowest level to make it more accessible to the decision-making system module or to adjust control parameters for guidance and actuation.
3. In the highest control level, the decision-making algorithm takes the information from the lower-level modules, and based on the desired behavior of the fully autonomous agricultural system, a plan to be executed by the guidance control and the treatment application is formulated.

After minimizing the hardware of the individual mobile units, the next step is to minimize the hardware of the whole fleet. The procedure of minimizing the hardware of a fleet of robots relies on the other elements that constitute the fleet of robots: the base station and the operator. As indicated in previous sections, the operator must be present at some instants of the application to configure and supervise the mission. Thus, an operator interface is essential, which can be provided in the form of a base station (computer monitor and keyboard) or a portable device (e.g., tablet, smartphone) that allows the operator to move close to the mobile units. A step forward in the configuration of the fleet of robots would be to structure a fully unmanned fleet with no operator intervention. This prospective case, which is not currently allowed by the legislation of many countries, would dispense with the base station, and the mission manager and the fleet supervisor would be run in the computing system of the mobile units. Two solutions are envisaged as follows.

### 5.1. Master-Slave Configuration

**Figure 12:**
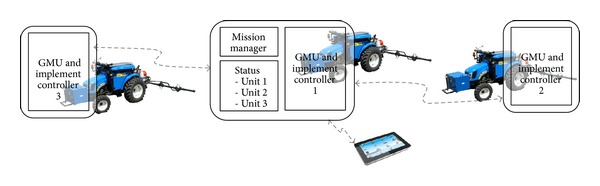
Master-slave configuration.

One fleet unit controller acts as a master running the mission manager and the supervisor of the fleet, while the rest acted as slaves receiving commands and returning data. A failure in this master controller also stops the mission of the fleet, but the likelihood of failure decreases because the whole fleet has one less computer and communication system (see Figure 12) with respect to the central-external controller solution. Adaptation to this topology is straightforward: the mission manager algorithms running on the base station computer can be packaged into a DLL and included, with minor software modifications, in a Main Controller, which will act as the fleet master controller. This process is the one explained in Figure 9.

Besides reducing hardware and structural elements in the fleet of robots, another advantage of this topology is the extension of the working area of the fleet. If the mission supervisor is fixed at a point in the field, the maximum working range of each unit is limited by the range of the communication system. A typical wireless network can have an open field range of up to 150 meters. As indicated in previous sections, the use of a fleet of autonomous robots in agriculture may be feasible in extensive applications that require long hours of continuous working in fields of tens of hectares. Thus, a larger communication range is required. One solution is the use of larger antennas and increasing the power of the transmitter/receiver to maintain an acceptable bandwidth or the use of signal repeaters. However, as there is a master unit in this topology, the mission supervisor has the ability to move around the field, which increases the working area of the entire fleet (maintaining a typical wireless network configuration), as long as the mission is defined so that each unit is within communication range.

### 5.2. Immerse Configuration

**Figure 13:**
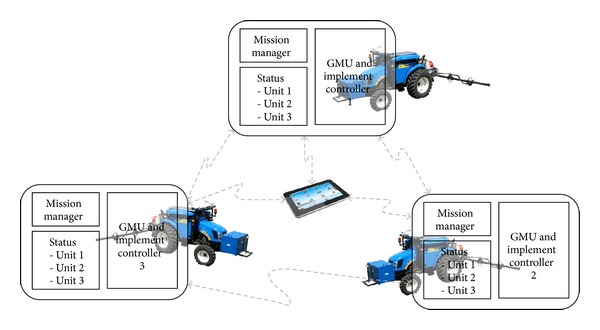
Immerse configuration.

The mission manager is copied in all mobile unit controllers so that a failure in one unit means that unit stops, while the rest can reconfigure the mission plan to accomplish the task. Note that the hardware is not increased and that the same mission manager algorithms run on every unit controller (see Figure 13), while the unit statuses are shared among all the robots by broadcasting a few status data in a sampling period basis. When a unit goes out of order, the others receive that information in the status or by a sampling period timeout; in such a case, the remaining active units will compute the mission manager, taking into account that incidence.

For this solution, there is not a clear gain of hardware reduction in the general architecture, but the immerse controller increases the robustness of the system by using a mirrored mission planner on each mobile unit controller. This immerse controller allows each mobile unit to supervise (mission supervisor) the execution of the plan and monitor the status (position, speed, etc.) of the other mobile units while adapting the missions of individual units to meet the goal of the fleet. This configuration, which is illustrated in Figure 13, is well suited to the hardware architecture presented in Section 5.1 for the ground mobile unit, in which the use of a cRIO system as the Main Controller permits direct communication with other cRIO systems without the development of drivers and communication protocols, thanks to the ability of the LabVIEW utilities to share information between NI systems.

## 6. Results

To present the implementation of a working fleet of robots configured with the Main Controller proposed in this paper, a set of assessments was conducted in a real experimental field as part of the RHEA project [40]. Several tests and integrations have been conducted that have positively assessed the system efficiency and ease of new integrations, which are organized as follows: Sections 6.1 and 6.2 present both quantitative and qualitative results associated with both hardware element reduction and software development minimization in a single, fully autonomous agricultural system; Section 6.3 presents the results of an algorithm for collision avoidance, allowing the assessment of the benefit of hardware reduction in a fleet of robots oriented to agriculture.

### 6.1. Integration of the Weed Detection System in the Main Controller

The first assessment trial was focused on evaluating both the image acquisition and processing procedure of the Weed Detection System by using the proposed architecture (see Figure 10) and compared them with those obtained with the original RHEA scheme (see Figure 7). For that trial, we measured the time required for each topology (centralized versus distributed) to acquire an image and generate an output, which was received in the Main Controller (see Table 3). In the first trial (with the original scheme), the computer was exclusively dedicated to image acquisition and image-processing tasks. However, using the proposed architecture, image acquisition, image processing, and four additional tasks defined in Table 3 were executed in parallel, meeting the scheduled time. Considering that each topology generates very similar results, we can conclude that we have maintained the required performance of the system, decreasing the hardware and developing a small number of communication interfaces. Furthermore, the image acquired by the Weed Detection System is available within the Main Controller in half the time using the architecture illustrated in Figure 10 compared to the original scheme (see Figure 7). Because the images are of high resolution, this time is quite significant when performing real-time calculations, and thus the same image can be shared with other processes, such as the obstacle detection system, avoiding redundant hardware (several cameras, for instance).

### 6.2. Integration of the Ground Mobile Unit Controller in the Main Controller

One more evaluation of the system was performed by removing the ground mobile unit controller (GMUC) in charge of the vehicle guidance and implementing path follower algorithms in the Main Controller. In this case, we evaluated the system capabilities to react to changes in both the trajectory and speed of the vehicle, which were measured as the number of messages sent to control both the vehicle speed and steering. Leaving aside the vehicle mechanical response and the performance of the path-following algorithms, by using the original RHEA scheme, the Main Controller can send messages (new trajectories) at a rate of 6–10 Hz. However, by using the proposed architecture, the Main Controller can send messages (new steering and speed references values) at a rate of 100 Hz. It is not correct to directly compare these two values because the messages correspond to diverse control levels. Therefore, a qualitative analysis must be performed. The original RHEA scheme defines the guidance system as a deliberative architecture in which the trajectory planning is performed by the Main Controller (based on a predefined mission and information of the perception system) and the GMUC executes that plan. The proposed architecture changes this configuration into a hybrid architecture, where, in critical situations (e.g., obstacle avoidance, row guidance, safety procedures), the capabilities of changing the position and orientation of the vehicle are improved. Although the behaviors of these two schemes are well known and have been studied for years [51, 52], they remain a current research topic [53] and are well suited to the requirements defined in Section 4.

The implemented controller relies on a fuzzy logic algorithm developed in [54].

### 6.3. Implementation of a Collision Avoidance Algorithm in the RHEA Fleet of Robots

As a final test to validate the use of the proposed architecture in a fleet of robots oriented to agricultural tasks, the implementation of a method for avoiding collisions between units was evaluated. The fleet configuration was as follows.

- Regarding each individual, fully autonomous agricultural system, the positioning system (RTK-GPS) was the only sensory element enabled for this test, in addition to the communication system (wireless communication) and the Main Controller (in charge of executing the mission and communicating with both the user and the mission supervisor).
- The fleet topology used in these tests was the master-slave configuration (see Figure 11), in which the algorithms to configure and transmit the mission to each unit, in addition to the fleet supervisor algorithm, were executed within the Main Controller onboard the GMU1. These algorithms had a built-in user interface that could be accessed remotely by the user via an external computer connected to the network of the fleet. With this interface the user can (a) monitor the status and location of each unit; (b) load a predefined mission to each unit; (c) record the GPS relative position of each unit; and (d) start, stop, or pause the motion of each unit, among other actions.

**Figure 14:**
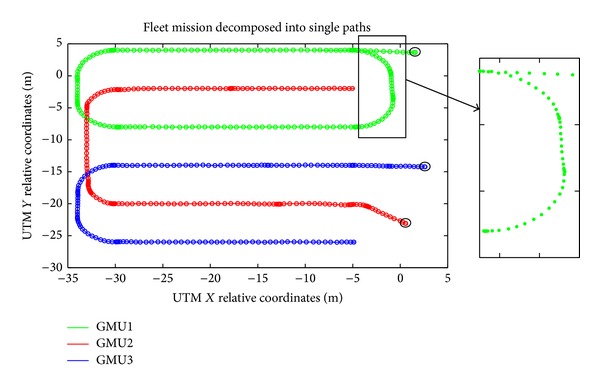
GPS position recorded for each unit representing the mission execution. The black circles represent the origin point of each unit.

Each unit must follow a user-predefined path at a constant speed. Each path consists of crossing a real field (35 meter long by 25 meter wide), making a U-shape turn, and returning down the field on a different crop line. The units are allowed to make the turns in an area with a length of approximately 8 meters at the headlines. The GPS positions recorded by each unit and the general mission sent to the fleet of robots are illustrated in Figure 14. Higher concentrations of GPS positions in the figure (the colored circles for each unit) indicate that the unit was moving at low speed or even stopped; by contrast, a lower concentration of GPS positions corresponds to normal development of the submission, following a predefined path at a constant speed.

Although the fleet mission can be defined as optimal both in time and space (because the characteristics of the field and the fleet are well known), it is possible to identify random external elements that alter the planned development of the mission and generate potential collision situations accordingly. Examples of these situations include the following: (a) detection of moving obstacles (e.g., persons, animals, and other tractors); (b) treatment parameters that affect the speed of operation (e.g., in a weed control treatment in which a decrease in speed is required for a more optimal application); (c) small temporary failures in the system itself (e.g., loss or decrease of the accuracy of the GPS signal, wireless communication loss).

**Figure 15:**
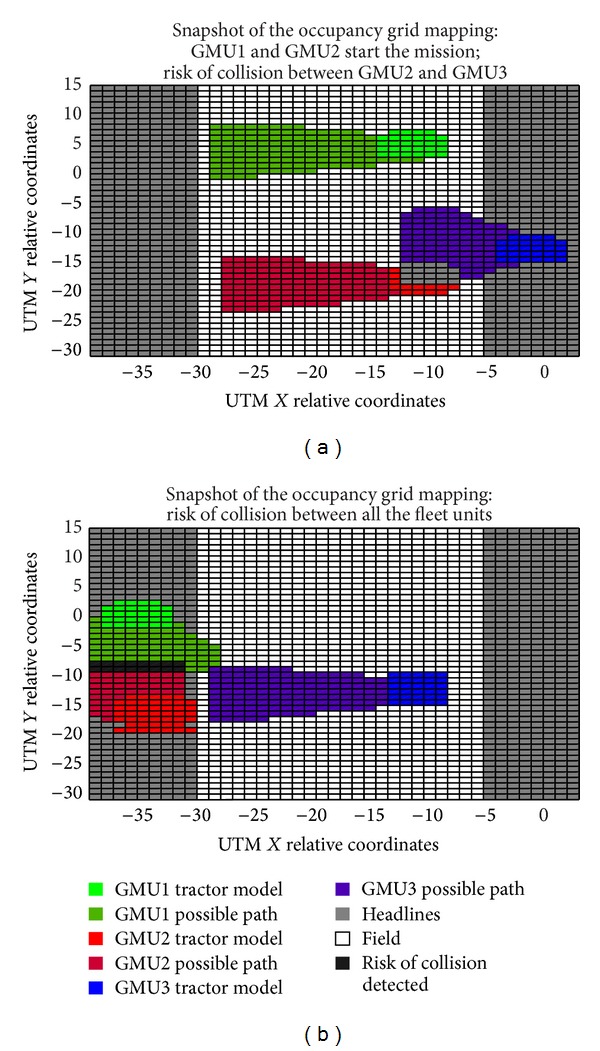
Snapshots of the occupancy grid mapping for collision detection. (a) Results of the collision detection within 10 seconds of the execution time of the mission. (b) Results of the collision detection within 25 seconds of the execution time of the mission.

To avoid collisions between units, the fleet supervisor algorithm contains a procedure that receives the GPS positions of each unit and calculates their possible location in subsequent time instants based upon their intended movement (current heading). The collision avoidance algorithm models each tractor as a square element and its intended motion as a conic section in which the vertex of the cone is in the center of each tractor. The opening angle of the conic section depends on whether the tractor is inside the field (smaller angle) or in the headlines (bigger angle), given that inside the field each tractor normally moves along a straight line. The fleet supervisor assigns priorities for each unit to continue its submission or stop until the risk of collision disappears. For this particular case, GMU1 has the highest priority, while GMU3 has the lower priority. The method for detecting potential collisions is occupancy grid mapping (see Figure 15).

**Figure 16:**
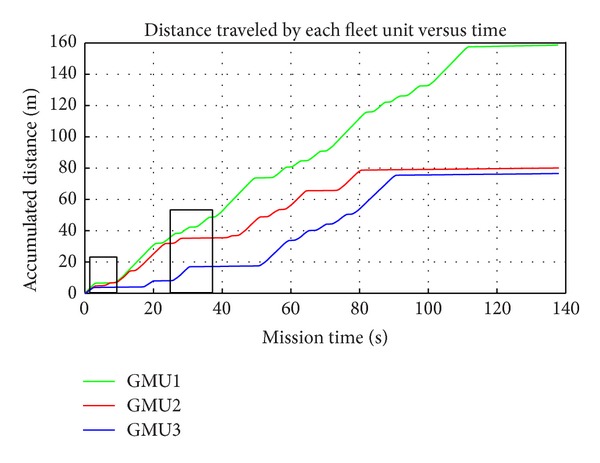
Accumulated distance traveled by each fleet unit as a function of the mission time.

Figure 16 illustrates the distance traveled by each fleet unit as a function of time. At some times (e.g., in the first 20 seconds of the mission; between the second 25 and 40 seconds), some units remain stopped because the fleet supervisor paused the execution of the submission of these units because there was a potential collision situation.  Figure 15(a) shows the result of the collision detection algorithm for an instant of time between the first 20 seconds of the general mission, during which a possible collision between GMU2 and GMU3 is present, and thus GMU3 will take longer to get to the other end of the field. This is the situation presented in Figure 15(b), in which GMU1 and GMU2 are making the turn to return to the field and another possible collision situation is present. In this situation, the fleet supervisor allows GMU1 to continue with its submission while stopping the movement of GMU2 until the collision situation disappears. In addition to the tests conducted for collision avoidance using the master-slave configuration, tests were performed with the original RHEA project configuration (see Figure 6), and as expected, the same results were obtained. These results confirm the potential of the proposed control architecture for an autonomous fleet of robots to allow hardware and software development reduction while maintaining the desired performance.

A video of the RHEA fleet is available at http://www.car.upm-csic.es/fsr/gds/RHEA_fleet.mp4eg; the video includes images of the user interface described in Section 6.3 as well as a real-time result of the collision avoidance algorithm.

## 7. Conclusions

Robotics and new technologies have begun to improve common practices in agriculture, such as increasing yield performance and decreasing the use of chemicals that may affect the environment. Furthermore, new robotics systems for application in agriculture are under development to permit the integration of different technologies while enabling modularity, flexibility, and adaptability.

This paper presents a structure for agricultural vehicles to work both independently and in fleets, that is, simple, robust, and reliable. The general scheme exhibits advantageous features to quickly implement new vehicle controllers and develop/integrate advanced agricultural implements. Three examples are reported herein: a boom sprayer, a mechanical-thermal machine, and an airblast sprayer.

The proposed architecture for the centralization of the Main Controller and the principal sensory systems provides some advantages for a future sensor fusion. Integrating critical sensors in autonomous agricultural applications, such as high-definition cameras and lasers systems, allows the information to be merged to improve the performance of the sensory system in terms of greater accuracy, greater robustness, and increased complementary data and to reduce the amount of hardware, which increases the communication speed and the information shared by different modules.

In addition, in an autonomous agricultural application, when the environment exhibits changing light, soil, and crop characteristics, among other characteristics, the sensory system is required to perform more complex tasks, which consequently leads to the problem of overcharging the Main Controller due to both the execution of multiple tasks in the same controller and the high consumption of resources for sensory fusion tasks. Nevertheless, in the proposed solution, this overuse is compensated by the Main Controller characteristics and its ability to execute diverse processes in parallel and in real-time as well as the possibility of implementing very specific and time-critical operations in the FPGA device.

This study attempts to increase the robustness of autonomous agriculture robots and fleets of robots by reducing the equipment hardware onboard the mobile units and facilitating the integration of different sensors devices and software modules developed by professionals in different fields and skills. Moreover, minimizing user involvement in monitoring and safety functions and enabling the same elements of the fleet to manage certain critical situations can also permit the reduction of the amount of hardware and structural elements in the fleet, which might increase the working area of the entire fleet.

The system is operational, and both individual and fleet robot features have been tested. The previous section illustrates two examples of subsystem integration into the Main Controller regarding the vision system and the vehicle controller, indicating quantitative features (see Table 3 and Section 6.2). Moreover, algorithms to allow the robots in the fleet to collaborate, follow a plan, and avoid collisions between robots by using the master-slave configuration have been presented in Section 6.3. In general, the proposed system has been assessed as very efficient to easily integrate new sensors, implements, and innovative algorithms in a fleet of agricultural robots.

The industrial exploitation of the fully unmanned fleet concepts presented in this paper is not yet permitted by the legislation of most countries. Nevertheless, the use of autonomous vehicles on public roads is under consideration in Japan, Sweden, and several states in the USA, and autonomous cars will unquestionably be allowed everywhere in the near future. In any case, the authorization of autonomous vehicles for closed scenarios such as farms will definitely occur first, and researchers are preparing for this eventuality.

## Figures and Tables

**Table 1:** Examples of autonomous vehicles for agricultural applications developed around the world.

Author/Centre	Blackmore et al., 2004. Dept. of Agricultural Sciences, Frederiksberg, Denmark [24]
Application	Automatic steered tractor capable of following a predefined route plan
Sensorial System	RTK GPS: Localization.
Results	The automatic steered tractor can follow a predetermined route within a few centimeters

Author/Centre	Cho and Lee, 2000. Department of Agricultural Engineering, Seoul National University, Korea [55]
Application	Autonomous operation of a speedsprayer in an orchard (a speedsprayer is defined as a power sprayer used to apply a highly concentrated pesticide in highly dispersed form by delivering it into a strong air blast generated by fans or blowers—Merriam-Webster Dictionary)
Sensorial System	DGPS for Localization; ultrasonic sensor for obstacle detection
Results	Speedsprayer autonomous operation: within 50-cm deviation. The speedsprayer could avoid trees or obstacles in emergency situations

Author/Centre	Hague et al., 2000. Silsoe Research Institute, Wrest Park, UK [56]
Application	Ground-based sensing methods for vehicle-position fixing
Sensorial System	Sensor package: machine vision, odometers, accelerometers, and a compass
Results	Reducing the low noise level of the odometric data and eliminating drift using sensor fusion

Author/Centre	Subramanian et al., 2006. Department of Agricultural and Biological Engineering, University of Florida, USA [57]
Application	Autonomous guidance system for use in a citrus grove
Sensorial System	Machine vision and laser radar (LADAR)
Results	Machine vision guidance: average error of 2.8 cm. LADAR guidance: average error of 2.5 cm (Tested in a curved path at a speed of 3.1 m/s)

Author/Centre	Xue et al., 2012. Department of Agricultural and Biological Engineering, University of Illinois, USA [58]
Application	Variable field-of-view machine vision method for agricultural robot navigation between rows in cornfields
Sensorial System	Machine vision with pitch and yaw motion control
Results	Maximum guidance error of 15.8 mm and stable navigational behavior

**Table 2:** Examples of autonomous implements for agricultural applications developed around the world.

Author/Centre	Blasco et al., 2002. Instituto Valenciano de Investigaciones Agrarias (IVIA), Spain [59]
Application	Non-chemical weed controller for vegetable crops
Sensorial System	Two machine vision systems: one in front of the robot for weed detection; the other for correcting inertial perturbations
Results	The system was able to eliminate 100% of small weeds. The system properly located 84% of weeds and 99% of lettuces

Author/Centre	Lee et al., 1999. Biological and Agricultural Engineering, University of California, USA [60]
Application	Real-time intelligent robotic weed control system for selective herbicide application to in-row weeds
Sensorial System	Two machine vision systems: one in front of the robot for guidance; the other for weed detection
Results	24.2% of the tomatoes were incorrectly identified and sprayed, and 52.4% of the weeds were not sprayed

Author/Centre	Leemans and Destain, 2007. Gembloux Agricultural University, Belgium [61]
Application	Positioning seed drills relative to the previous lines while sowing
Sensorial System	Machine vision for guidance
Results	The standard deviation of the error was 23 mm, with a range of less than 100 mm

Author/Centre	Pérez-Ruiz et al., 2012. University of California, Davis, Department of Biological and Agricultural Engineering, USA [8]
Application	Automatic mechanical intra-row weed control for transplanted row crops
Sensorial System	RTK-GPS for controlling the path of a pair of intra-row weed knives
Results	A mean error of 0.8 cm in centering the actual uncultivated close-to-crop zone about the tomato main stems, with standard deviations of 1.75 and 3.28 cm at speeds of 0.8 and 1.6 km/h, respectively

**Table:** (a)

Time required	Image Acquisition	Fps acquired	Image Processing	Fps processed	Image Sharing	Other process running
Original structure	75–150 ms	5	150–250 ms	4	150–200 ms	0
Proposed structure	80–160 ms	5	200–250 ms	4	1 ms	4 (See Table 3(b) for process description)

**Table:** (b)

Other process running	Scheduled periods
Path following supervising routine	100 ms
Steering and throttle control routine	10 ms
Telemetry routine	100 ms
Localization routine	100 ms
